# Prevalence and Factors Associated with Anxiety and Depression in Peruvian Medical Students

**DOI:** 10.3390/ijerph20042907

**Published:** 2023-02-07

**Authors:** Jorge L. Piscoya-Tenorio, Walter V. Heredia-Rioja, Noelia Morocho-Alburqueque, Sandra Zeña-Ñañez, Palmer J. Hernández-Yépez, Cristian Díaz-Vélez, Virgilo E. Failoc-Rojas, Mario J. Valladares-Garrido

**Affiliations:** 1 School of Medicine, Universidad Nacional Pedro Ruiz Gallo, Lambayeque 14013, Peru; 2 Sociedad Científica de Estudiantes de Medicina de la Universidad Nacional de Piura (SOCIEMUNP), Piura 20007, Peru; 3 School of Medicine, Universidad Continental, Lima 15046, Peru; 4 South American Center for Education and Research in Public Health, Universidad Norbert Wiener, Lima 15046, Peru; 5 School of Medicine, Universidad Privada Antenor Orrego, Trujillo 13008, Peru; 6 Hospital Nacional Almanzor Aguinaga Asenjo, EsSalud, Chiclayo 14001, Peru; 7 Unidad de Investigación para Generación y Síntesis de Evidencia en Salud, Universidad San Ignacio de Loyola, Lima 15024, Peru; 8 Oficina de Epidemiología, Hospital Regional Lambayeque, Chiclayo 14012, Peru

**Keywords:** anxiety, depression, mental health, medicine students

## Abstract

During medical training, students are exposed to stressors that deteriorate mental health. This study determined depression and anxiety prevalence and their associated factors in students from four medical schools in northern Peru. A cross-sectional study was conducted among medical students from Lambayeque, Peru. The Goldberg Anxiety and Zung Depression tests were applied. The dependent variables were depression and anxiety, and their association with covariates (age, sex, type of university, socioeconomic level, experience, family problem, and physical activity) was evaluated. Prevalence ratios were calculated using generalized linear models. Of 482 students, the prevalence of anxiety was 61.8% and depression was 22.0%. A severe level of anxiety was observed in 6.2% in the group between 16 and 20 years old. It was observed that private university students had higher frequencies of having depression (PR = 2.01) and anxiety (PR = 1.35); males had a lower risk of anxiety (PR = 0.82), but higher risk of depression compared to females (PR = 1.45). Performing physical activity decreased the prevalence of depression (PR = 0.53); however, it increased the frequency of anxiety (PR = 1.26). Having family problems increased the prevalence of anxiety (PR = 1.26). Medical students from private universities experienced higher rates of depression and anxiety. Depression and anxiety were associated with gender and physical activity. These findings highlight the importance of mental health promotion due to its link with quality of life and academic performance.

## 1. Introduction

Medical students are exposed to various stressors, which deteriorates their mental health within medical training [1,2,3]. The global prevalence of symptoms of anxiety and depression in health science students is high. Around 30% of students experience some form of mental disorder [4,5,6,7]. However, these figures could be higher and more heterogeneous in Latin American countries [8], including Peru, where a prevalence higher than 60% has been reported [9,10,11,12]. These high estimates are attributed to various intrinsic and extrinsic factors [13]. In this context, several studies have demonstrated the existence of risk factors, such as female sex [14], academic overload [15], socio-economic level [1,5], and protective factors such as year of study [16], spirituality [17], motivation [2], resilience, and other coping strategies [15].

Although there is evidence in this regard, some limitations can be recognized in the literature. In addition to the inherent subjective bias resulting from the “self-reported” nature of data collection [13], the cross-sectional design of many studies limits their ability to establish causality between variables [13,15]. On the other hand, some investigations have been conducted with a small sample of students from a single educational center [14,18]. Likewise, there are some factors that have been little explored [5], such as family history, physical activity, mode of living, and year of study. This study seeks to investigate these little-explored variables, including a larger sample of medical students and enhancing the identification of the study population variability using a multicenter approach.

This study determined depression and anxiety prevalence and their associated factors in students from four medical schools in northern Peru.

## 2. Materials and Methods

### 2.1. Study Design

A cross-sectional multicenter study was conducted in medical students during September–November 2019.

### 2.2. Population and Sample

The population consisted of medical students from 4 universities in the Lambayeque region, located in northern Peru. The target population was all medical students of the 4 universities, which was 5800, based on the students enrolled during the semester in which the study was conducted. OpenEpi v.3.01 was used to determine the sample size, estimating a 60% expected proportion by background, an absolute precision of 5%, a confidence level of 95%, and a design effect of 1.3 (due to stratification of universities), resulting in a minimum sample size of 451 students. A random sampling was carried out in each university, reaching 482 students distributed as follows: 240 from the Universidad Nacional Pedro Ruiz Gallo, 109 from the Universidad Particular de Chiclayo, 89 from the Universidad de San Martín de Porres, and 44 from the Universidad Señor de Sipán.

### 2.3. Instrument

The instrument was a questionnaire that included three sections (see Supplementary Material). The first consisted of general socio-educational information, measured by self-report, such as age (years), sex (female, male), socioeconomic level (What is your socioeconomic level? Low, medium, high), type of university (public, private), people with whom the student lives (with family, alone), family problems (Did you have family problems during the last semester? No, yes), and physical activity (Do you perform physical activity? No, yes). 

The second section included the Goldberg scale. This scale is useful for detecting symptoms of anxiety. This scale is composed of nine dichotomous response questions, where each affirmative response is assigned a point, and a point of ≥ 4 is considered as presence of anxiety [19]. Higher values indicate more symptom severity, with 9 points as the highest scale value. Reivan-Ortiz et al., in their study, found that the reliability determined by Cronbach’s alpha coefficient was 0.80, concluding that the scale met reliability criteria [20]. Likewise, Martin-Carbonell found sensitivity values of 71% and a specificity of 77% [21].

The last section included the Zung Self-Assessment Scale for Depression. It is a short self-assessment survey consisting of 20 questions where symptoms related to depressive episodes are explored (mood state, with two items, and cognitive and somatic symptoms, each with eight items, and two psychomotor symptoms); there are ten questions formulated in a positive way and another ten formulated in a negative way. Each question uses a four-point Likert scale, with four response options: 1 (very rarely), 2 (sometimes), 3 (frequently), and 4 (almost always). A global score of 40 or more is considered as depressive symptoms with clinical significance [22]. Novara et al. reported a reliability coefficient of 0.75 [23].

### 2.4. Variables

The dependent variables were depression and anxiety. Depression was defined with the Zung Self-Assessment Scale when a score of 40 or more points was obtained. Anxiety was defined with an average score ≥ 4 points from the Goldberg scale.

The independent variables were categorized as age (categorized: 16 to 20, >20), sex (female, male), type of university (public, private), economic level (low, medium, high), self-reported having family problems (no, yes), self-reported physical activity (no, yes), and living alone/accompanied

### 2.5. Study Procedure

The selected medical students from each university were explained the objective of the study and provided with details of the collection instrument and the time for completion, emphasizing that the survey would be anonymous and completion would be voluntary. Immediately afterwards, they were given the printed questionnaire, which they filled out in writing and individually. The data obtained were entered into the Microsoft Excel program.

### 2.6. Statistical Analysis

Analyses were carried out with STATA 15.0. Frequencies and percentages were reported for categorical variables. For categorical variables, Chi-square or Fischer’s exact tests were applied to estimate the association between depression and anxiety and socio-educational variables, after assessing statistical assumptions. In the simple regression analysis, generalized linear models (GLM), Poisson distribution family, log link function, and robust variance were used to investigate the factors associated with depression and anxiety. Prevalence ratios (PR) and 95% confidence intervals were estimated. We considered *p* values <0.05 as statistically significant.

### 2.7. Ethical Considerations

The study protocol was reviewed and approved by the ethics committee of the Universidad Nacional Pedro Ruiz Gallo, with file N° CIEI-FM-UNPRG-017-2019. Informed consent was provided to participants, highlighting that the study was voluntary and anonymous. Participants’ confidentiality was preserved using assigned codes.

## 3. Results

Table 1 shows the socio-educational characteristics of the students. A total of 482 students were surveyed. The proportion of sex was similar, and it was observed that students older than 20 years made up 60.2%. A large percentage belonged to middle socioeconomic level (86.3%) and also had family problems (64.5%).

Table 2 shows the bivariate analysis. It was observed that students in private universities had higher frequencies of having depression (29.3% vs. 14.6%) and anxiety (71.1% vs. 52.5%) compared to students in public universities; males were less likely to have anxiety (56.0% vs. 68.8%), but more likely to develop depression (25.8% vs. 17.8%) compared to females.

Table 3 shows the simple regression results. Students from private universities had higher frequencies of having depression (PR = 2.01) and anxiety (PR = 1.35); males had a lower risk of anxiety, but a higher risk of depression compared to females. Likewise, having family problems increased the probability of having anxiety by 26% with respect to those who did not have family problems (PR = 1.26, 95%CI: 1.04–1.53, *p* = 0.019); physical activity decreased the probability of having depression by 47% compared with individuals not performing physical activity (PR = 0.53, 95%CI: 0.34–0.81, *p* = 0.002).

## 4. Discussion

### 4.1. Prevalence of Mental Health Disorders

Almost one out of five students experienced depression in universities from Lambayeque. Previous studies corroborate this result [24,25,26]. This is consistent with the results of Sanchez-Marín et al. (2016), who found that 17.3% of students presented some depressive episode [27]. In Peru, 13.5% were found to have some degree of depression [28]. However, this differs from the result of Vilchez-Cornejo (2016), who reported a prevalence of depression in 32.5% of students [29]. These differences could have occurred because different measurement scales were used (Zung and DASS-21, respectively) and because they were conducted in three different departments of Peru.

Almost three out of five students experienced anxiety at universities in Lambayeque. This finding is consistent with that documented in other studies [30,31]. Likewise, Kulsoom et al. (2015) found that 63% of medical students presented anxiety [32]. However, it differs from that evidenced by Jafari et al. (2017), where 78.33% of students presented anxiety, and by Lemos et al. (2018), where the percentage was 48.3%. [33,34]. In both cases, the differences could be due to the context where the studies were conducted, Iran and Colombia, respectively.

### 4.2. Factors Associated with Mental Health Disorders

We found that attending a private university increases the prevalence of depression and anxiety. Previous studies reported similar results [35,36]; however, in Bangladesh [37] and Nigeria [38], it was found that students from public universities had higher estimates. The association between studying at a private university and depression and anxiety could be explained by the pressure on students to repay high payments from their parents or funders by obtaining high grades.

Students older than 20 years had a 17% lower prevalence of anxiety. Similarly, Carbonell et al. (2019) showed that lower anxiety was associated with higher student age [31]. Likewise, in Peru, students in their third or fourth year had a lower frequency of anxiety than those in early academic years [39]. However, in China, students older than 20 years had higher depression and anxiety estimates [40]. This could be because younger students, as they are finishing adolescence and beginning early adulthood, are in a period of vulnerability for the development of mental health problems, added to the process of adaptation to a medical career [41].

Male students had 45% higher prevalence of depression than female students. In contrast, males had 18% lower anxiety prevalence. Likewise, two systematic reviews showed that female students had higher anxiety levels [5] and male students had higher depression levels [1]. However, previous studies have shown opposite results [16,35], where higher levels of depression and anxiety were found in the female sex. These differences may be due to the period in which the questionnaire was taken (e.g., after exams) and to the different populations evaluated and different scales used. In Latin America, depression was also associated with being female [14] and, in Peru, female sex was associated with 1.03 times higher risk of depression, but this was not statistically significant [42].

Having family problems increases anxiety prevalence by 26%. Obregón-Morales (2020) reported a similar result, showing that having a dysfunctional family increased, by more than two times, the probability of having depression [42]. Shao et al. (2020) reported that social support and family functioning were associated with symptoms of depression and anxiety [40]. This could be explained because family social support is key to coping with depression, and dysfunctional families would not be prepared to exercise this support, which would increase depressive symptoms in students [43,44]. On the other hand, in those who lived alone, depression and anxiety levels were higher [40].

Physical activity was associated with a 47% decrease of depression prevalence. Conversely, it increases, by 26%, the prevalence of anxiety. Likewise, Carbonell et al. (2019) showed that a greater number of weekly sports hours was considered a protective factor for depression, but also for anxiety [31]. De Souza et al. (2021) reported that deficient physical activity was linked to greater symptoms of depression and anxiety [45]. However, another study [46] found no significant association between physical activity and depression or anxiety, although data were collected within the context of the COVID-19 pandemic, so other factors may have played a role. It is reported that adults should perform regular physical activity to have a lower risk of depression [47]. This is supported by the fact that exercise generates physiological changes such as increased endorphin levels and maintenance of mitochondrial function and of the neurotransmitters serotonin, dopamine, and noradrenaline, which contribute to improved mood and decreased stress [48,49,50].

### 4.3. Limitations and Strengths

This research has limitations. First, non-probabilistic sampling was performed, so there may be a possible selection bias. Second, some of the variables, such as family problem and physical activity, were measured by self-report, so it would not be known if the considerations were real. Third, there may be some residual confounding because covariates that could influence depression and anxiety in medical students were not explored; for example, smartphone use and its relationship with mental health outcomes [51]. Fourth, the study design limits the establishment of causality. Fifth, we applied an exploratory epidemiological approach, performing only unadjusted analysis. To perform multiple regression analysis, the type of relationship between variables should be identified through a more sophisticated theoretical model (e.g., classifying confounders and mediators by a directed acyclic graph). Therefore, we suggest performing studies with confirmatory epidemiological approaches based on the present results. Despite these limitations, the main strength of the present study lies in its quality of recognizing that there are characteristics associated with anxiety and depression among medical students in the first university years, which may guide and strengthen wellness programs related to the preservation of mental health in medical students.

## 5. Conclusions

Medical students from private universities experienced a higher prevalence of depression and anxiety. Depression and anxiety prevalence were associated with gender and physical activity. These findings highlight the importance of mental health promotion among medical students because it influences their quality of life and academic well-being.

## Figures and Tables

**Table 1:** Socio-educational characteristics of medical students from Lambayeque, Peru.

Characteristics	n (%)
**Type of university**	
Public	240 (49.8)
Private	242 (50.2)
**Age in years (categorized)**	
16 to 20	192 (39.8)
>20	290 (60.2)
**Sex**	
Female	230 (47.7)
Male	252 (52.3)
**Socioeconomic level**	
Low	65 (13.5)
Medium	416 (86.3)
High	1 (0.2)
**Living conditions**	
With family	43 (8.9)
Alone	439 (91.1)
**Family problems**	
No	171 (35.5)
Yes	311 (64.5)
**Physical activity**	
No	322 (66.8)
Yes	160 (33.2)
**Depression**	
Normal	376 (78.0)
Mild	100 (20.7)
Moderate	5 (1.0)
Severe	1 (0.2)
**Anxiety**	
Normal	184 (38.2)
Mild	152 (31.5)
Moderate	96 (19.9)
Severe	50 (10.4)

**Table 2:** Factors associated with depression and anxiety in medical students in Lambayeque, Peru.

Variables	Depression		*p* *	Anxiety		*p* *
	No (n = 376)	Yes (n = 106)		No (n = 184)	Yes (n = 298)	
	n (%)	n (%)		n (%)	n (%)	
**Type of university**			**<0.001**			**<0.001**
Public	205 (85.4)	35 (14.6)		114 (47.5)	126 (52.5)	
Private	171 (70.7)	71 (29.3)		70 (28.9)	172 (71.1)	
**Age in years (categorized)**			0.783			**0.011**
16 to 20	151 (78.7)	41 (21.4)		60 (31.3)	132 (68.8)	
> 20	225 (77.6)	65 (22.4)		124 (42.8)	166 (57.2)	
**Sex**			**0.035**			**0.005**
Female	189 (82.2)	41 (17.8)		73	157 (68.3)	
Male	187 (74.2)	65 (25.8)		111 (44.1)	141 (56.0)	
**Socioeconomic level**			0.163			0.263
Low	45 (69.2)	20 (30.8)		21 (32.3)	44 (67.7)	
Medium	330 (79.3)	86 (20.7)		162 (38.9)	254 (61.1)	
High	1 (100.0)	0 (0.0)		1 (100.0)	0 (0.0)	
**Living conditions**			0.343			0.642
With family	36 (83.7)	7 (16.3)		15 (34.9)	28 (65.1)	
Alone	340 (77.5)	99 (22.6)		169 (38.5)	270 (61.5)	
**Family problems**			0.215			**0.019**
No	128 (74.9)	43 (25.2)		113 (53.6)	98 (46.5)	
Yes	248 (79.7)	63 (20.3)		71 (41.5)	100 (58.5)	
**Physical activity**			**0.002**			**0.001**
No	238 (73.9)	84 (26.1)		139 (43.2)	183 (56.8)	
Yes	138 (86.3)	22 (13.8)		45 (28.1)	115 (71.9)	

* *p*-values calculated with the Chi-Square test of independence.

**Table 3:** Analysis of factors associated with depression and anxiety in medical students in Lambayeque, Peru.

Characteristics	Simple Regression					
	Depression			Anxiety		
	PR	95% CI	*p* *	PR	95% CI	*p* *
**Type of university**						
Public	Ref.			Ref.		
Private	2.01	1.40–2.89	**<0.001**	1.35	1.17–1.56	**<0.001**
**Age in years (categorized)**						
16 to 20	Ref.			Ref.		
> 20	1.05	0.74–1.48	0.783	0.83	0.73–0.96	**0.011**
Sex						
Female	Ref.			Ref.		
Male	1.45	1.02–2.05	**0.035**	0.82	0.71–0.94	**0.006**
**Socioeconomic level**						
Low	Ref.			Ref.		
Medium	0.67	0.45–1.01	0.068	0.90	0.75–1.08	0.306
High						
**Living conditions**						
With family	Ref.			Ref.		
Alone	1.39	0.69–2.79	0.343	0.94	0.75–1.19	0.642
**Family problems**						
No	Ref.			Ref.		
Yes	0.81	0.57–1.13	0.215	1.26	1.04–1.53	**0.019**
**Physical activity**						
No	Ref.			Ref.		
Yes	0.53	0.34–0.81	**0.002**	1.26	1.10–1.45	**0.001**

* *p*-values obtained with Generalized Linear Models (GLM), Poisson family, log-link function, and robust variance.

## Data Availability

The dataset generated and analyzed during the current study is not publicly available because the ethics committee has not provided permission/authorization to publicly share the data but are available from the corresponding author on reasonable request.
